# A scoping review of strategies to support public health recovery in the transition to a “new normal” in the age of COVID-19

**DOI:** 10.1186/s12889-022-13663-2

**Published:** 2022-06-23

**Authors:** Emily Belita, Sarah E. Neil-Sztramko, Alanna Miller, Laura N. Anderson, Emma Apatu, Olivier Bellefleur, Lydia Kapiriri, Kristin Read, Diana Sherifali, Jean-Éric Tarride, Maureen Dobbins

**Affiliations:** 1 School of Nursing, McMaster University, 1280 Main St. West, HSC 2J22, Hamilton, ON L8S 4K1 Canada; 2 Department of Health Research Methods, Evidence, and Impact, McMaster University, 175 Longwood Road South, Suite 210a, Hamilton, ON L8P 0A1 Canada; 3 National Collaborating Centre for Methods and Tools, McMaster Innovation Park, 175 Longwood Road South, Suite 210a, Hamilton, ON L8P 0A1 Canada; 4 Department of Health Research Methods, Evidence, and Impact, McMaster University, 1280 Main St. West, Hamilton, ON L8S 4K1 Canada; 5 Centre de collaboration nationale sur les politiques publiques et la santé (CCNPPS), National Collaborating Centre for Healthy Public Policy (NCCHPP) , 190, boulevard Crémazie Est, Montréal, Québec H2P 1E2 Canada; 6 Department of Health, Aging & Society, McMaster University, 1280 Main St. W. KTH 236, Hamilton, ON L8S 4M4 Canada; 7 School of Nursing, McMaster University, 1280 Main Street West , Hamilton, ON L8S 4K1 Canada; 8 School of Nursing, McMaster University, National Collaborating Centre for Methods and Tools , 175 Longwood Road South, Suite 210a, Hamilton, ON L8P 0A1 Canada

**Keywords:** Pandemic, COVID-19, Disaster, Public health system, Disaster recovery

## Abstract

**Background:**

During the COVID-19 pandemic, the public health workforce has experienced re-deployment from core functions such as health promotion, disease prevention, and health protection, to preventing and tracking the spread of COVID-19. With continued pandemic deployment coupled with the exacerbation of existing health disparities due to the pandemic, public health systems need to re-start the delivery of core public health programming alongside COVID-19 activities. The purpose of this scoping review was to identify strategies that support the re-integration of core public health programming alongside ongoing pandemic or emergency response.

**Methods:**

The Joanna Briggs Institute methodology for scoping reviews was used to guide this study. A comprehensive search was conducted using: a) online databases, b) grey literature, c) content experts to identify additional references, and d) searching reference lists of pertinent studies. All references were screened by two team members. References were included that met the following criteria: a) involved public health organizations (local, regional, national, and international); b) provided descriptions of strategies to support adaptation or delivery of routine public health measures alongside disaster response; and c) quantitative, qualitative, or descriptive designs. No restrictions were placed on language, publication status, publication date, or outcomes. Data on study characteristics, intervention/strategy, and key findings were independently extracted by two team members. Emergent themes were established through independent inductive analysis by two team members.

**Results:**

Of 44,087 records identified, 17 studies were included in the review. Study designs of included studies varied: descriptive (*n* = 8); qualitative (*n* = 4); mixed-methods (*n* = 2); cross-sectional (*n* = 1); case report (*n* = 1); single-group pretest/post-test design (*n* = 1). Included studies were from North America (*n* = 10), Africa (*n* = 4), and Asia (*n* = 3) and addressed various public health disasters including natural disasters (*n* = 9), infectious disease epidemics (*n* = 5), armed conflict (*n* = 2) and hazardous material disasters (*n* = 1). Five emergent themes were identified on strategies to support the re-integration of core public health services: a) community engagement, b) community assessment, c) collaborative partnerships and coordination, d) workforce capacity development and allocation, and e) funding/resource enhancement.

**Conclusion:**

Emergent themes from this study can be used by public health organizations as a beginning understanding of strategies that can support the re-introduction of essential public health services and programs in COVID-19 recovery.

**Supplementary Information:**

The online version contains supplementary material available at 10.1186/s12889-022-13663-2.

## Background

Public health crises place significant burden on local health departments who are responsible for leading emergency responses; previous disease outbreaks have showcased substantial impacts of disaster response on public health staffing capacity and service availability [1–4]. Health departments are primarily tasked with implementing essential public health services [3] within core functions of health protection, health surveillance, disease and injury prevention, population health assessment, health promotion, and emergency preparedness and response [5]. Essential or core public health programs/services represent the minimum level of services public health entities provide within a public health system [6, 7] Essential or core programs/services address issues at the primordial, primary, or secondary prevention levels and prevent diseases/conditions which are potential and critical health threats [6]. They also aim to improve the health and resilience of populations and represent interventions for which there is a reasonable evidence base of their effectiveness [6]. Given international contextual variation, there are various perspectives on specific essential or core programs/service. However, examples across different countries have included immunization, chronic disease prevention, environmental health [8, 9], substance use, and school health [10]. Complexity emerges when in disaster response, some essential services may be compromised and health departments must make critical internal adaptations to staffing (e.g., reallocation, surge capacity), function, and organizational structure to divert resources appropriately [1, 3, 11].

The COVID-19 pandemic provides a clear and recent example of a major disruption to health systems across the world. While the focus has been primarily on frontline workers in acute-care medicine, the public health workforce has also been affected [12]. With the extensive deployment of the public health workforce from core functions (i.e., health promotion, disease prevention, and health protection), to a focus on preventing and tracking the spread of COVID-19 through case and contact tracing and mass vaccination, public health no longer looks the way it did in March 2020 [13, 14]. A well-functioning public health system is critical to the health of a population [15, 16]. As pandemic deployment continues, there is an urgent need for public health systems to resume delivery of core public health programming alongside COVID-19 activities [13] to prevent exacerbation of other health risks and disparities within populations [17, 18].

Emergency preparedness plans for a pandemic have long existed in public health and include key activities to align with distinct emergency management phases [19]. The response phase includes activities during or immediately before or after a disaster that address its direct impacts (e.g., emergency medical assistance) [20]. The recovery phase of an emergency/disaster includes repair or restoration activities to support the return to a pre-disaster circumstance. Activities may include the provision of public health and safety programs, restoration of suspended essential services, re-establishment of infrastructure, provision of basic necessities of food, water, shelter for displaced individuals, and the protection of environmental resources [20]. Lessons learned from the recovery of past emergencies, albeit on smaller scales, can be used to inform the development of much needed supports for the public health workforce and related organizations [19, 21]. However, the longer the deployment, the more challenging this will be. As the public health system and workforce begins to pursue recovery strategies from COVID-19, it will be necessary to identify evidence-informed solutions to meet the emerging challenges public health and its workforce will face in restructuring and reintroducing critical public health programs.

To date, existing scoping reviews on emergency preparedness have focused on describing general principles for public health emergency preparedness [22], general attributes of resilient health care systems [16], and mapping the status and nature of the existing evidence base on public health emergency preparedness [23]. A scoping review, describing and prioritizing 42 general preparedness planning and evaluation recommendations identified seven key domains among the reviewed literature: 1) governance, 2) capacity building and maintenance, 3) surveillance, 4) assessment, 5) risk and crisis management, 6) post-event evaluation, and 7) implementation of lessons learned [22]. Post-event-related recommendations which concentrated primarily on evaluation, had the fewest number of established recommendations [22]. In another scoping review of 77 key documents (scholarly articles and grey literature), [16] 16 themes of health system resilience were identified in the context of infectious disease and natural hazard disasters. While findings speak to the need for post-event recovery plans addressing several specific issues (e.g., re-establishing social networks, response plan revision), results did not explicitly describe or recommend strategies to be used in these plans. This review [16] also underscores the absence of implementation frameworks to operationalize attributes of resilient systems into public health practice. An additional recent scoping review mapped the current public health emergency preparedness evidence by exploring content themes and identifying knowledge gaps [23]. In this review, of 300 articles analyzed in relation to the emergency management cycle, only 3% focused on disaster recovery, while 42% focused on the immediate response phase. A key knowledge gap identified from this review and accompanying stakeholder consultation was the theme of resilience, specifically, what role public health plays in supporting recovery and return to more resilient and equitable circumstances [23].

Existing reviews provide a general understanding about core elements of public health system preparedness and highlight that there is limited literature on disaster recovery practices and approaches [24, 25]. This is important to note given public health plays an essential role in disaster recovery. Public health organizations are involved in both short and long-term recovery activities which may include supporting continuity of health services, assessing environmental infrastructure, identifying specific needs of marginalized groups, leading surveillance to issue public health advisories and communication to the mass public [26]. Furthermore, the existing synthesized evidence does not specifically address re-integration strategies in post-disaster circumstances.

## Methods

The aim of this scoping review was to identify existing strategies to support re-introduction of core public health programming alongside ongoing pandemic or emergency response within the public health sector. Given existing reviews have validated that the disaster recovery literature is limited, a scoping review was identified as the most suitable type of review to provide a beginning understanding of the existing landscape. This scoping review was guided by the Joanna Briggs Institute’s methodology for scoping reviews [27].

### Search strategy

The search strategy was developed in consultation with a Health Sciences Librarian. An initial search was conducted on 10 August 2020 and an updated search on 2 November 2020. Six databases were searched from inception including: MEDLINE, Embase, EMCARE, PsycINFO, CINAHL, Business Source Premier and ABI/INFORM (see Additional file 1 for search example). Additionally, we conducted a grey literature search using the Canadian Agency for Drugs and Technologies in Health grey literature checklist and additional websites identified by research team members, contacted various field experts, and manually searched the reference lists of pertinent studies to identify additional published and unpublished studies not identified in the scientific databases. All identified references were uploaded to DistillerSR and duplicates were removed. Two team members independently screened all titles and abstracts against the established inclusion and exclusion criteria. Potentially relevant full-text citations were retrieved and screened in duplicate by two team members for final inclusion. Discrepancies were resolved through discussion, and when required, a third team member arbitrated the final inclusion decision.

### Eligibility criteria and study selection

Studies were included in the review if they met the following criteria: a) involved public health organizations (e.g., health departments, health authorities), including local, regional, national and international organizations, and organizations that work collaboratively with public health, such as social service and community organizations; b) provided descriptions of strategies to support public health organizations adapt or deliver routine public health measures alongside response; and c) were conducted during or after a public health emergency (i.e., occurrences or threat of substantial damage, injury, or loss of life/property due to human acts or natural events [28]) that necessitated a rapid and systemic emergency response (e.g., disease outbreak, pandemic, natural disaster, warfare). Any primary research articles, including randomized controlled trials, quasi-experimental studies, cohort studies, case control studies, cross-sectional studies, case reports, qualitative studies and descriptive studies were eligible. Studies were excluded if: a) they were hospital or acute care-based studies without a public health component; b) addressed ongoing public health crises without distinct starting points or which did not necessitate a rapid response involving redeployment of resources; c) described strategies only in the context of immediate disaster response; and d) letters, commentaries or articles based solely on opinion. No restrictions were placed on language, publication status, date of publication, or outcomes.

### Data extraction

Data extraction was completed in DistillerSR using a standardized data extraction form. The data extraction form was piloted on three included studies and revisions were made to the form following pilot testing. For each study, extracted data included the authors, year of publication, country, the name, type, length, and year of each public health emergency identified, study design and objectives, funding source, involved organizations, time between disaster onset and study, target audience of interventions, program type, description of the intervention, and main findings. Two authors extracted data in duplicate from each reference and discrepancies were resolved through discussion.

### Data synthesis

We conducted a descriptive numerical analysis of study characteristics and qualitative analysis of themes [29]. We analyzed frequencies of study characteristics according to study design, country and continent of origin, disaster type, public health services offered alongside disaster response, and stakeholder involvement. Regarding strategies to support the re-integration of public health services, we conducted qualitative inductive thematic analysis [29, 30] in duplicate by two members of the research team. Discrepancies were resolved through consensus. We analyzed strategies according to disaster type to determine trends among specific types of disasters (e.g., infectious disease epidemic, natural disaster).

## Results

**Fig. 1:**
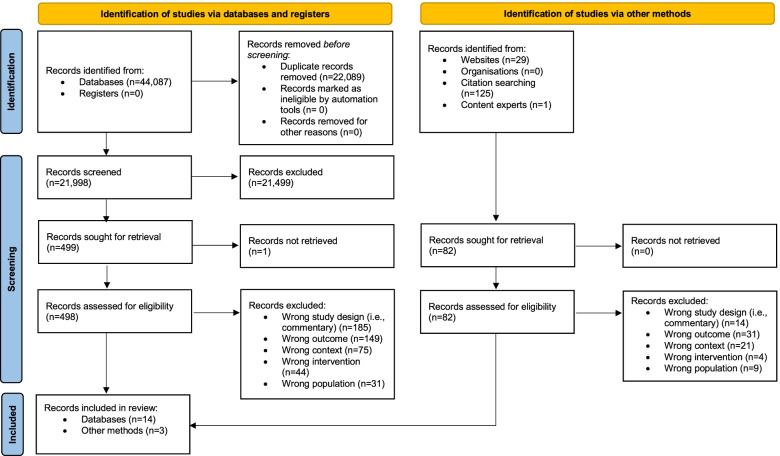
PRISMA

The database search retrieved 44,087 records. An additional 155 references were identified through supplemental search strategies (websites, citation searching, content experts). After duplicates were removed, 21,998 titles and abstracts were screened, and 580 records were reviewed in full (498 identified via databases/registers and 82 identified via other methods). Seventeen articles were included in the final review, 14 from databases and three from other methods (see Fig. 1 PRISMA).

### Characteristics of studies

Included studies were published from 2005 and comprised a variety of designs: descriptive (*n* = 8); qualitative (*n* = 4); mixed-methods (*n* = 2); cross-sectional (*n* = 1); case report (*n* = 1); single-group pretest/post-test design (*n* = 1). Many of the included studies were from North America including the United States (*n* = 8) and Haiti (*n* = 2). Four studies originated from Africa including West Africa (*n* = 3) and Uganda (*n* = 1). Three studies were from Asia, with one study each originating from Burma, Thailand, and Yemen. Included studies addressed a variety of public health disasters, including natural disasters (*n* = 9), infectious disease epidemics (*n* = 5), armed conflict (*n* = 2) and hazardous material disasters (*n* = 1).

Studies discussed the provision of diverse services and activities during disaster recovery which addressed several public health areas including infectious diseases (*n* = 8), nutrition (*n* = 6), maternal and infant health (*n* = 5), general emergency preparedness and response (*n* = 5), parenting and child development (*n* = 3), immunizations (*n* = 3), sexual health (*n* = 2), chronic diseases (*n* = 2), substance use (*n* = 1), physical activity (*n* = 1), and environmental health and safety (*n* = 1). These services and activities were implemented by a variety of service providers, including local health departments (*n* = 8), state/provincial health authorities (*n* = 6), federal agencies (*n* = 5) non-governmental organizations (*n* = 13) and other organizations (*n* = 6). Most of the studies described services that were provided by multiple types of service providers (see Supplementary File 2 for detailed characteristics of included studies).

Strategies for re-integration of public health services/programs.

Across 17 included studies, five emergent themes were identified. Studies often discussed multiple strategies (see Table 1).

**Table 1:** Five emergent themes for pandemic recovery identified from 17 included studies

Emergent Themes	Number of studies	Study Citations
1. Collaborative partnerships (local, state, global) and coordination	11	[31–41]
2. Community assessment	7	[33–36, 39, 40, 42]
3. Workforce capacity development, allocation	7	[34, 35, 37–41]
4. Community engagement	5	[43–47]
5. Funding/resource enhancement	4	[35, 37, 38, 41]

The total number does not add to 17 since some studies were categorized under multiple themes

‘Collaborative partnerships and coordination’ was an emergent theme identified in 11 studies [31–41]. These studies described the integral role of partnerships between multi-sectoral and multi-level (local, state, international) players in post-disaster activities. Across some studies, formal partnership structures such as large scale coalitions [31, 33, 34, 40], local committees, regional hubs or networks [32, 33, 35, 36, 38, 40] involving public health organizations were instituted to mobilize and facilitate the coordination of physical or human resources for recovery purposes. Key stakeholders varied across recovery activities, although common players included public health organizations (local and state level), international bodies (e.g., World Health Organization, United Nations), government (state or federal) and non-government agencies, academic institutions, private sector businesses, and local community health and social service organizations. Collaborative activity among these partners involved coordinated approaches to community needs assessments, strategic recovery planning to map out partner activities, and agreements on the allocation of shared resources and implementation of services and programs to prevent duplication [31, 34, 37, 38, 41]. Collaborative partnerships and coordination among these studies addressed diverse and multiple needs in relation to infectious diseases (*n* = 5), maternal/infant health (*n* = 4), general emergency preparedness and response (*n* = 3), parenting (*n* = 2), nutrition (*n* = 3), immunizations (*n* = 2), and sexual health (*n* = 2).

‘Community assessment’ was identified as a theme in seven studies [33–36, 39, 40, 42]. Health-related needs and priorities were identified by directly engaging local community residents in assessments [34–36, 42] and relying on the expertise of local stakeholders [33, 40]. Formal, structured assessments to determine evolving health status and needs during recovery periods were conducted using national surveys [34], epidemiological methodologies such as the Community Assessments for Public Health Emergency Response (CASPER) [36, 42], and surveillance systems (Geographic Information System) [34] to support recovery planning, decision-making and to initiate responsive public health programming/services. Implementation and use of data from these regular, structured assessments allowed for monitoring the progression of recovery processes through tracking of diverse indicators related to demographics, post-disaster experiences, physical and behavioural health status, mental health, vaccination rates, or disease-specific indicators. Unstructured or informal community assessments were also conducted to collect local anecdotal community data [36]. Needs assessments also encompassed an analysis of the public and health care system to determine gaps in infrastructure, human and financial resources that would be needed to support recovery interventions [35, 39]. While studies stated that collected data were used to guide prioritization and decision-making around emergent needs, details on the explicit processes used to do so were not elaborated on.

‘Workforce allocation and capacity development’ emerged from seven studies [34, 35, 37–41]. These studies reported on the use of continuous training opportunities to maintain workforce competency, and strategies employed to increase human resource capacity in recovery activities. Five studies described the establishment of training programs that aimed to develop knowledge and skills in diverse areas of disaster preparedness and recovery [35], general epidemiological principles (e.g., surveillance, data collection/analysis) [34], specific communicable diseases (e.g., Ebola) [41], chronic disease prevention [37], or population-specific issues (i.e., childhood immunization, maternal/child care) [40]. Training programs targeted diverse workforce groups including local volunteers [41], community health workers (CHWs) [34, 37, 40], and local community leaders [35]. Strategies discussed to address workforce gaps included the establishment of tiered volunteer systems [35, 38], creation of temporary health centers or teams of staff to provide preventive health services during short-term recovery [38, 39], the use of professional students or new graduates (nursing or midwifery) [38, 40], and assuming roles outside of practice scope (e.g., midwives serving as vaccinators or general health educators) [40]. A critical point in sustaining workforce capacity emphasized by two studies involved the preservation of workforce psychological well-being [39, 41]. Geiger et al. [41] discussed strategies to address this including task shifting for fatigue prevention, contingency plans to cover absences due to illness and the implementation of psychosocial teams to support psychological well-being.

‘Community engagement’ emerged as a predominant theme in five studies [43–47]. Community-engaged strategies reflected a strength-based approach in which the social capital of impacted communities was utilized. The implementation of CHW programs was reported in four studies [43–45, 47]. Across these studies, CHWs were residents from impacted communities who had diverse professional backgrounds in social work, health care (e.g., nursing), education, in addition to youth and staff with non-profit community organization experience [43, 45]. CHWs conducted a myriad of health promotion, protection, and prevention activities in disaster/epidemic recovery periods including relationship building, home visits, referral generation and follow-up, needs assessment and screening, individual health counselling, linking to community resources and organizations, and leading community groups (e.g., exercise or nutrition sessions). CHW programs targeted specific populations (e.g., maternal/child health) [47], specific communicable diseases (e.g., tuberculosis prevention post-Ebola epidemic) [44, 45], or chronic disease prevention and management [43]. Comprehensive training to ensure CHW competency [43–45] and fair financial compensation were reported as important facilitators in CHW program implementation [47]. One additional study reported on the use of a community-based participatory service approach to address community health-related concerns in a post-disaster circumstance and help sustain longer-term recovery processes [46]. At the centre of this approach was the establishment of a community coalition of local volunteer leaders that championed and organized townhall meetings to identify priorities and needs, training workshops for community members on various public health topics, the establishment of a community health tracking registry, and the implementation of health screenings conducted by community volunteers [46].

‘Funding and resource enhancement’ was identified as a theme among four studies [35, 37, 38, 41]. These studies emphasized the critical nature of establishing long-term and flexible funding frameworks to sustain resources required for programs or services over the course of longer recovery periods instead of short-term funding mechanisms. Craddock et al. [35] discussed the importance of advanced planning to secure long-term recovery funding through diverse means including donations, proposals/grants, and formal funding allocations in annual healthcare and public health budgets. State of emergency declarations and the establishment of new governmental policies were reported as facilitating supplemental funding to hire additional public health personnel for surging recovery demands [38].

Trends also emerged when mapping specific strategies to disaster type (see Table 2). Community engagement was employed as a strategy across all four disaster types related to infectious diseases, natural disasters, armed conflict, and hazardous material. Across the natural disaster literature (*n* = 9) all five strategies were represented, with most studies discussing community assessment (*n* = 6) and collaborative partnerships (*n* = 7). Among the studies addressing infectious disease epidemics (*n* = 5), almost all strategies were discussed, except for community assessment. Infectious disease literature was consistently distributed across the strategies of community engagement (*n* = 2), collaborative partnerships (*n* = 3), workforce capacity (*n* = 3), and funding/resource enhancement (*n* = 3).

**Table 2:** Number of included studies according to recovery strategy and disaster type

Strategy Theme	Type of Disaster (# of studies) [citations]			
	**Infectious disease epidemic (*n* = 5)**	**Natural disaster (*n* = 9)**	**Armed conflict (*n* = 2)**	**Hazardous material (*n* = 1)**
1. Community engagement (*n* = 5)	(*n* = 2) [44, 47]	(*n* = 1) [43]	(*n* = 1) [45]	(*n* = 1) [46]
2. Community assessment (*n* = 7)	–-	(*n* = 6) [33–36, 39, 42]	(*n* = 1) [40]	–-
3. Collaborative partnerships (local, state, global) and coordination (*n* = 11)	(*n* = 3) [37, 38, 41]	(*n* = 7) [31–36, 39]	(*n* = 1) [40]	–-
4. Workforce capacity development, allocation (*n* = 7)	(*n* = 3) [37, 38, 41]	(*n* = 3) [34, 35, 39]	(*n* = 1) [40]	–-
5. Funding/resource enhancement (*n* = 4)	(*n* = 3) [37, 38, 41]	(*n* = 1) [35]	–-	–-

## Discussion

This scoping review provides an understanding of common strategies used to support the re-integration of core public health services during disaster recovery. The limited and largely descriptive evidence on recovery strategies identified in our review coincides with findings from a recent scoping review which characterized the general state and nature of public health emergency preparedness and response (PHEPR) research according to the Centers for Disease Control and Prevention’s 15 PHEPR capabilities [48]. Testa et al. [48] identified 1,106 articles; most were categorized as “community preparedness” (21.9%) with a focus on pre-disaster planning or immediate response. In comparison, only 6.1% of included articles, predominantly descriptive in nature, discussed some aspect of “community recovery” involving post-disaster recovery operations, risk communication, needs assessment or rebuilding community infrastructure [48]. Findings from our scoping review support the notion that there is a general paucity of literature centred on public health crisis recovery, and of the existing studies, most do not assess strategy or intervention effectiveness [23, 25].

Across the community recovery evidence identified in the scoping review by Testa et al. there [48] was a large emphasis on post-disaster needs assessments. Included studies consisted primarily of cross-sectional surveys to collect data on community health outcomes [48]. This finding aligns with the theme of community assessment emerging from our review. Our review identifies the use of diverse mechanisms including surveys, interviews, and surveillance systems to assess health status indicators and highlights the importance of system capacity assessments to guide recovery decision-making and action. The Institute of Medicine [24] further acknowledges that short and long-term recovery require an evidence-informed approach utilizing already established community health assessments, large public health data sets, newly developed disaster-specific needs assessments, and health impact assessments. Notably, community assessment was commonly discussed in the context of natural disasters and not infectious disease epidemics. Although examples from our review such as the use of epidemiological methodologies like the Community Assessments for Public Health Emergency Response (CASPER) [42] may be useful in pandemic instances, further evaluation of this is needed. Needs assessments are a critical first step in COVID-19 recovery given growing health disparities among populations and reduced public health prevention and control measures to address chronic diseases and mitigate the impacts of inequities related to the social determinants of health; they provide vehicles for prioritization of services and programs of those in most need [13]. There was also limited discussion across the literature on how data-driven prioritization and decision-making are operationalized in recovery.

Tools for prioritization and continuity planning of essential services have been discussed in the context of immediate pandemic responses [49, 50], with potential to adapt, implement, and evaluate these products and processes in a recovery context. In these instances, strategic and multi-step processes have been used which include a first step of convening a steering committee or working group to lead assessment and service prioritization [49, 50]. Assessment includes an analysis of health status/threats, system capacity, community engagement, and political context. This is followed by identification and prioritization of all services provided by the public health organization into three categories: a) essential services/functions (time sensitive and disruption would cause harm or loss of life); b) services that contribute to health outcomes and can be suspended temporarily; and c) services that can be suspended for longer periods of time [49]. A last step includes developing a model of services while considering resources, required workforce skill set and capacity, and impact of model implementation [49].

Other emergent themes in our review are consistent with recommendations and guiding principles established by expert organizations, coalitions/committees that have been convened to disseminate information related to disaster recovery practices. The Institute of Medicine [24] discusses the importance of community engagement in disaster recovery efforts. Authentic engagement requires that impacted communities are involved in all phases of a recovery project, from the first step of identifying needs to selecting priorities and implementing programs [24]. Findings from our review reflect different structures and degrees of community engagement, from the use of a local community coalition to lead broad scale needs assessments and prioritization to the implementation of CHW programs. Our findings concentrate primarily on the role diversity of CHWs in disaster recovery which include both communicable and non-communicable disease activities. The use of CHWs has been similarly discussed in a rapid review of 36 studies [51] which found that CHW programs have been implemented primarily in low and middle-income countries in the immediate COVID-19 response. CHW roles centred on awareness-raising and contract tracing although some CHWs provided adapted essential routine services. Our findings also align with the Resilience Framework for Public Health Emergency Preparedness developed by Khan et al. [52] which includes community engagement as an essential element for public health systems in disaster response and recovery. In this framework, community engagement involves the mobilization of community assets, establishment of communication and knowledge connections and an understanding that engagement differs and evolves according to context and different disaster phases [52]. Within this contextual understanding, considerations need to be factored in with respect to policy, legal, and regulatory requirements in engaging community participants in recovery efforts.

In their proposed disaster recovery recommendations, the Institute of Medicine also calls for a coordinated approach that addresses integrated planning and implementation across different sectors, levels of government, and throughout evolving phases of a disaster [24]. This parallels the theme of collaborative partnerships and coordination from our review that includes a description of both large- and small-scale partnerships with diverse stakeholders and activities. These results relate well to the attribute of collaborative and coordinated networks, an essential element for resilient public health systems against emergencies and disasters [16, 52]. This attribute includes establishing role clarity, infrastructure, and partnering with health and non-health sectors to leverage expertise and resources. Vital to efficient and coordinated partnerships are trust, fortified relationships, and shared vision [24]. Successful networks must benefit from infrastructure establishment, cultivating of relationships and planning during a pre-disaster era which can be facilitated through the development of integrated emergency preparedness or business continuity plans and partnership agreements. While coordinated pre-disaster recovery planning has been recommended in guidance documents [24, 53], concrete examples of this did not emerge in our review, highlighting a need for further exploration around it.

Scoping review results concerning public health workforce capacity and funding also align with recovery strategies focused on resource enhancement recommended elsewhere [16, 24, 54]. Nyasulu et al. [54] propose concrete solutions for maintaining essential services alongside the COVID-19 pandemic that target human resource sustainment which include web-based training, task shifting, role expansion, and health care professional student engagement [54]. While not captured in our study findings, Nyasulu et al. [54] also expand on staff shortage discussions by recommending innovative strategies such as recruitment of qualified non-working health care professionals, converting part-time positions to full-time, and accelerating professional certification for senior health care students. Further exploration is needed on the use of these innovative workforce strategies and their ability to offset the needs of public health workforces experiencing burnout in an ongoing public health crisis. Similar to our findings, Khan et al. [52] underscore the importance of investing in training to facilitate an adequate, competent workforce, with expertise in public health content, emergency management and professional skills such as communication and collaboration to support workforce redundancy. Our review also discusses securing financial resources through different channels beyond government funding such as private sector donors or grants to fund recovery efforts. The emergence of this as a strategy may relate to the unique context of these studies (i.e., involvement of non-governmental organizations). As well, this strategy is commonly associated with disasters specifically impacting single geographic regions in which donations can be more easily solicited and distributed. This speaks to the importance of considering the context in the use of identified strategies from our review and the potential transferability given the type of disaster, breadth of impact, and partner involvement in recovery practices.

When analyzing disaster context, findings demonstrated that most studies addressed natural disasters as opposed to infectious diseases epidemics. This is a common finding among the general emergency preparedness and response literature which often features natural disasters occurring more than a decade ago with long recovery periods [24]. The Institute of Medicine [24] however, acknowledges that there is emerging research underway on more recent disasters. This is likely the case with the current COVID-19 pandemic given that recovery planning is actively underway and more time is needed to implement, author, and publish on recovery strategies. Our findings also support the case that more evidence is needed on the re-integration of core public health services specifically in the context of infectious disease emergencies. It is important to consider when interpreting our findings that the ways in which recovery strategies were implemented likely differed due to diverse contextual factors [24]. Studies included in our review originated from different geographic settings (North America, Africa, and Asia) with varying degrees of available resources and infrastructure, along with variable disaster types, which can influence the type of appropriate strategies for implementation.

While our review findings make important contributions to the literature, there are some limitations. Given the evidence base was largely descriptive with limited information on strategy outcomes, analysis and synthesis on outcomes or strategy effectiveness were not completed. This translates into a call to action for more rigorous research to be conducted on re-integration strategies in public health recovery that evaluates both process and impact. As well, most studies addressed natural disasters, which have different community impacts and public health responses compared to infectious disease epidemics or warfare conflict also featured in this review. As such, the transferability of findings requires consideration of context. Despite this, the challenge of competing priorities in public health disaster recovery transcends disaster types and provides at least a starting point for how to approach recovery given the limited evidence. Another challenge when conducting public health focused reviews is how to define public health organizations given international variability. In screening studies, we considered organizations which were involved in advancing any public health core function or providing essential/core programs/services. Within any review, it is also possible that relevant literature may have been missed. As a future strategy, the use of a formal stakeholder consultation to assist in developing the search terms and strategy will be considered. Notably, our team consulted with three content experts to support identification of missed literature and two experienced Business and Health Sciences librarians to develop a comprehensive search strategy.

## Conclusions

The evidence on strategies to support the re-integration of essential public health services alongside disaster response is limited and primarily descriptive in nature. The existing evidence however, does provide an intial understanding about re-integration strategies that include collaborative and coordinated efforts, assessing community needs and system capacity, enhancing human and financial resources, and engaging community residents. This knowledge provides a starting point for public health organizations as they begin recovery decision-making and processes for a post-COVID-19 era. Future research is needed to evaluate re-integration strategies in a variety of disaster, geographic and socio-economic contexts to develop a better understanding of the effective strategies in diverse settings.

## Supplementary Information

**Additional file 1.** Example of online database search – MEDLINE.

**Additional file 2.** Detailed Data Extraction.

## Data Availability

All data generated or analysed during this study are included in this published article [and its supplementary information files].

## Abbreviations

PHEPR: Public health emergency preparedness and response
CHW: Community health worker

## References

[1] Enanoria WTA, Crawley AW, Hunter JC, Balido J, Aragon TJ (2014). The epidemiology and surveillance workforce among local health departments in California: mutual aid and surge capacity for routine and emergency infectious disease situations. Public Health Rep.

[2] Schuh RG, Tony Eichelberger R, Stebbins S, Pomer B, Duran L, Mahoney JF (2012). Developing a measure of local agency adaptation to emergencies: a metric. Eval Program Plann.

[3] Potter MA, Schuh RG, Pomer B, Stebbins S (2013). The adaptive response metric: toward an all-hazards tool for planning, decision support, and after-action analytics. J Public Health Manag Pract.

[4] Shipp Hilts A, Mack S, Li Y, Eidson M, Nguyen T, Birkhead GS (2016). New York State public health system response to hurricane sandy: an analysis of survey feedback. Disaster Med Public Health Prep.

[5] Butler-Jones D (2008). The Chief Public Health Officer's report on the state of public health in Canada 2008: Addressing health inequalities.

[6] Population Health and Wellness Ministry of Health Services Province of British Columbia (2005). A framework for core functions in public health.

[7] National Association of County & City Health Officials (2012). Statement of Policy Minimum Package of Public Health Services.

[8] Lampe S, Atherly A, VanRaemdonck L, Matthews K, Marshall J (2015). Minimum package of public health services: the adoption of core services in local public health agencies in Colorado. Am J Public Health.

[9] Hoss A, Menon A, Corso L (2016). State public health enabling authorities: results of a fundamental activities assessment examining core and essential services. J Public Health Manag Pract.

[10] Ontario Ministry of Health and Long-Term Care (2018). Ontario Public Health Standards: Requirements for Programs, Services, and Accountability.

[11] Schuh RG, Basque M, Potter MA (2014). The effects of funding change and reorganization on patterns of emergency response in a local health agency. Public Health Rep.

[12] Gupta N, Balcom SA, Gulliver A, Witherspoon RL (2021). Health workforce surge capacity during the COVID-19 pandemic and other global respiratory disease outbreaks: a systematic review of health system requirements and responses. Int J Health Plann Manage.

[13] Brownson RC, Burke TA, Colditz GA, Samet JM (2020). Reimagining public health in the aftermath of a pandemic. Am J Public Health.

[14] Edmonds JK, Kneipp SM, Campbell L (2020). A call to action for public health nurses during the COVID-19 pandemic. Public Health Nurs.

[15] Haldane V, De Foo C, Abdalla SM, Jung A-S, Tan M, Wu S (2021). Health systems resilience in managing the COVID-19 pandemic: lessons from 28 countries. Nat Med.

[16] Nuzzo JB, Meyer D, Snyder M, Ravi SJ, Lapascu A, Souleles J (2019). What makes health systems resilient against infectious disease outbreaks and natural hazards? Results from a scoping review. BMC Public Health.

[17] Rozenfeld Y, Beam J, Maier H, Haggerson W, Boudreau K, Carlson J (2020). A model of disparities: risk factors associated with COVID-19 infection. Int J Equity Health.

[18] Bambra C, Riordan R, Ford J, Matthews F (2020). The COVID-19 pandemic and health inequalities. J Epidemiol Community Health.

[19] Tam TWS (2020). Preparing for uncertainty during public health emergencies: what Canadian health leaders can do now to optimize future emergency response. Healthc Manage Forum.

[20] Emergency Management Policy and Outreach Directorate Public Safety Canada (2017). An emergency management framework for Canada.

[21] Rosella LC, Wilson K, Crowcroft NS, Chu A, Upshur R, Willison D (2013). Pandemic H1N1 in Canada and the use of evidence in developing public health policies–a policy analysis. Soc Sci Med.

[22] Belfroid E, Roβkamp D, Fraser G, Swaan C, Timen A (2020). Towards defining core principles of public health emergency preparedness: scoping review and Delphi consultation among European Union country experts. BMC Public Health.

[23] Khan Y, Fazli G, Henry B, de Villa E, Tsamis C, Grant M (2015). The evidence base of primary research in public health emergency preparedness: a scoping review and stakeholder consultation. BMC Public Health.

[24] Institute of Medicine (2015). Healthy, Resilient, and Sustainable Communities After Disasters: Strategies, Opportunities, and Planning for Recovery.

[25] National Academies of Sciences E, Medicine (2020). Evidence-Based Practice for Public Health Emergency Preparedness and Response. Calonge N, Brown L, Downey A, editors.

[26] Walsh L, Garrity S, Rutkow L, Thompson CB, Strauss-Riggs K, Altman BA (2015). Applying a behavioral model framework for disaster recovery research in local public health agencies: a conceptual approach. Disaster Med Public Health Prep.

[27] Peters MDJ, Godfrey C, McInerney P, Munn Z, Tricco AC, Khalil H. 2020. JBI Manual for Evidence Synthesis. Joanna Briggs Institute.

[28] Haffajee R, Parmet WE, Mello MM (2014). What is a public health “emergency”?. N Engl J Med.

[29] Levac D, Colquhoun H, O'Brien KK (2010). Scoping studies: advancing the methodology. Implement Sci.

[30] Braun V, Clarke V (2006). Using thematic analysis in psychology. Qual Res Psychol.

[31] Fitter DL, Delson DB, Guillaume FD, Schaad AW, Moffett DB, Poncelet JL (2017). Applying a new framework for public health systems recovery following emergencies and disasters: the example of Haiti following a major earthquake and cholera outbreak. Am J Trop Med Hyg.

[32] Acosta JD, Burgette L, Chandra A, Eisenman DP, Gonzalez I, Varda D (2018). How community and public health partnerships contribute to disaster recovery and resilience. Disaster Med Public Health Prep.

[33] Quinn D, Lavigne SV, Chambers C, Wolfe L, Chipman H, Cragan JD (2008). Addressing concerns of pregnant and lactating women after the 2005 hurricanes: the OTIS response. MCN Am J Matern Child Nurs.

[34] Centers for Disease Control and Prevention (2013). Progress Toward Rebuilding Haiti’s Health System.

[35] Craddock HA, Walsh L, Strauss-Riggs K, Schor K (2016). From leaders, for leaders: advice from the lived experience of leaders in community health sector disaster recovery after Hurricanes Irene and Sandy. Disaster Med Public Health Prep.

[36] Kennedy M, Gonick S, Meischke H, Rios J, Errett NA (2019). Building back better: local health department engagement and integration of health promotion into hurricane harvey recovery planning and implementation. Int J Environ Res Public Health.

[37] Kodish SR, Simen-Kapeu A, Beauliere JM, Ngnie-Teta I, Jalloh MB, Pyne-Bailey S (2019). Consensus building around nutrition lessons from the 2014–16 Ebola virus disease outbreak in Guinea and Sierra Leone. Health Policy Plan.

[38] Stoto MA, Nelson C, Higdon MA, Kraemer J, Hites L, Singleton CM (2013). Lessons about the state and local public health system response to the 2009 H1N1 pandemic: a workshop summary. J Public Health Manag Pract.

[39] Melgaard B, Profili MC, Heimann P, Pusponegoro A, O'Rourke E, Kolokathis A (2005). Panel 2.9: repair and recovery of health systems. Prehosp Disaster Med.

[40] Tappis H, Elaraby S, Elnakib S, Alshawafi NAA, Basaleem H, Al-Gawfi IAS (2020). Reproductive, maternal, newborn and child health service delivery during conflict in Yemen: a case study. Confl Health.

[41] Geiger D, Harborth L, Mugyisha A (2020). Managing enduring public health emergencies such as COVID-19: lessons from uganda red cross society's ebola virus disease response operation. BMJ Leader.

[42] Schnall AH, Roth JJ, Ellis B, Seger K, Davis M, Ellis EM (2019). Addressing community needs during the hurricane response and recovery efforts through community assessments for public health emergency response (CASPER)-United States Virgin Islands, 2017–2018. Disaster Med Public Health Prep.

[43] Russell D, Oberlink MR, Shah S, Evans L, Bassuk K (2018). Addressing the health and wellness needs of vulnerable rockaway residents in the wake of hurricane sandy: findings from a health coaching and community health worker program. J Public Health Manag Pract.

[44] Rogers JH, Jabateh L, Beste J, Wagenaar BH, McBain R, Palazuelos D (2018). Impact of community-based adherence support on treatment outcomes for tuberculosis, leprosy and HIV/AIDS-infected individuals in post-Ebola Liberia. Glob Health Action.

[45] Lee CI, Smith LS, Shwe Oo EK, Scharschmidt BC, Whichard E, Kler T (2009). Internally displaced human resources for health: villager health worker partnerships to scale up a malaria control programme in active conflict areas of eastern Burma. Glob Public Health.

[46] Abara W, Wilson S, Vena J, Sanders L, Bevington T, Culley JM (2014). Engaging a chemical disaster community: lessons from Graniteville. Int J Environ Res Public Health.

[47] Miller NP, Milsom P, Johnson G, Bedford J, Kapeu AS, Diallo AO (2018). Community health workers during the Ebola outbreak in Guinea, Liberia, and Sierra Leone. J Glob Health.

[48] Testa M, Savoia E, Piltch-Loeb R, Biddinger P, Su M, Hayes J (2020). Review and Evidence Mapping of Scholarly Publications Within the CDC's 15 Public Health Emergency Preparedness and Response Capabilities.

[49] Global Health Cluster COVID-19 Task Team (2020). Essential health services: A guidance note: How to prioritize and plan essential health services during COVID-19 response in humanitarian settings.

[50] Niagara Region Public Health (2006). Pandemic influenza response plan: Business continuity planning toolkit.

[51] Bhaumik S, Moola S, Tyagi J, Nambiar D, Kakoti M (2020). Community health workers for pandemic response: a rapid evidence synthesis. BMJ Glob Health.

[52] Khan Y, O'Sullivan T, Brown A, Tracey S, Gibson J, Généreux M (2018). Public health emergency preparedness: a framework to promote resilience. BMC Public Health.

[53] Federal Emergency Management Agency (2016). National Disaster Recovery Framework.

[54] Nyasulu J, Pandya H (2020). The effects of coronavirus disease 2019 pandemic on the South African health system: a call to maintain essential health services. Afr J Prim Health Care Fam Med.

