# Supplementary material for: A scoping review of strategies to support public health recovery in the transition to a “new normal” in the age of COVID-19
Source: BMC Public Health. 2022 Jun 23;22:1244. doi: 10.1186/s12889-022-13663-2 (PMC9219400; doi:10.1186/s12889-022-13663-2)
Supplement: Supplementary file 1 — Additional file 1. Example of online database search – MEDLINE. [file 12889_2022_13663_MOESM1_ESM.docx]

**Additional File 1. Example of online database search – MEDLINE**

1 public health/

2 public health*.mp.

3 health department*.mp.

4 preventative medicine/

5 preventative medicine*.mp.

6 public health practice/

7 public health nursing/

8 community health services/

9 community health*.mp.

10 community health nursing/

11 health facility administrators/

12 population health*.mp.

13 preventative health*.mp.

14 disease outbreaks/

15 (disease outbreak* or infection outbreak*).mp.

16 epidemics/ or pandemics/

17 (epidemic* or pandemic*).mp.

18 space-time clustering/

19 disasters/ or disaster*.mp.

20 disaster planning/

21 strategic stockpile/

22 (strateg* adj2 stockpile*).mp.

23 health care rationing/

24 (health* adj2 ration*).mp.

25 natural disasters/

26 medical countermeasures/

27 contact trac*.mp.

28 Influenza A Virus, H1N1 Subtype/ or (H1N1 or swine flu*).mp.

29 SARS Virus/ or (severe acute respiratory syndrome or SARS).mp.

30 Middle East Respiratory Syndrome Coronavirus/ or (middle eastern respiratory syndrome or MERS).mp.

31 Hemorrhagic Fever, Ebola/ or ebola*.mp.

32 (corona* or covid*).mp.

33 terrorism/

34 "Warfare and Armed Conflicts"/ or Warfare/

35 (terroris* or armed conflict* or warfare or war).mp.

36 (national emergenc* or international emergenc* or humanitarian emergenc* or national cris* international cris* or humanitarian cris*).mp.

37 organizational innovation/

38 change management/

39 (organization* innovat* or change manage* or organization* change).mp.

40 "Organization and Administration"/

41 public health administration/

42 Social Control Policies/

43 models, organizational/

44 (organization* transform* or organization* reform* or organization* restructur* or organization* transition* or organization* model*).mp.

45 health planning/

46 delivery of health care/

47 ((deliver* adj2 health care*) or (deliver* adj2 healthcare*)).mp.

48 health planning guidelines/

49 health priorities/

50 Health Resources/

51 (health priorit* or health resource* or (resource* adj2 allocat*)).mp.

52 (health* system adj2 resilien*).mp.

53 Systems Analysis/

54 Workflow/

55 capacity building/ or staff development/

56 (build* adj1 capacit*).mp.

57 professional* develop*.mp.

58 ((staff* adj2 develop*) or (employ* adj2 develop*) or (staff adj2 train*) or (employee adj2 train*)).mp.

59 Occupational health/

60 "personnel staffing and scheduling"/

61 health workforce/ and (public health* or health department*).mp.

62 (health personnel* and (public health* or health department*)).mp.

63 health services administration/ and (public health* or health department*).mp.

64 ((health adj2 administrat*) and (public health* or health department*)).mp.

65 (maintain* adj2 (core service* or core function*)).mp.

66 (maintain* adj2 (essential service* or essential function*)).mp.

67 ((essential function* or core function*) and (public health* or health department*)).mp.

68 ((vaccin* or immuniz*) and (public health* or health department*)).mp.

69 (Operation* strateg* and (public health* or health department*)).mp.

70 (communicat* strateg* and (public health* or health department*)).mp.

71 (coordinat* mechani* and (public health* or health department*)).mp.

72 redeploy*.mp.

73 restruct*.mp.

74 21 or 22 or 23 or 24 or 37 or 38 or 39 or 40 or 41 or 42 or 43 or 44 or 45 or 46 or 47 or 48 or 49 or 50 or 51 or 52 or 53 or 54 or 55 or 56 or 57 or 58 or 60 or 61 or 62 or 63 or 64 or 65 or 66 or 67 or 69 or 70 or 71 or 72 or 73

75 1 or 2 or 3 or 4 or 5 or 6 or 7 or 8 or 9 or 10 or 11 or 12 or 13

76 14 or 15 or 16 or 17 or 18 or 19 or 20 or 25 or 26 or 27 or 28 or 29 or 30 or 31 or 32 or 33 or 34 or 35 or 36

77 74 and 75 and 76
