# Supplementary material for: A scoping review of strategies to support public health recovery in the transition to a “new normal” in the age of COVID-19
Source: BMC Public Health. 2022 Jun 23;22:1244. doi: 10.1186/s12889-022-13663-2 (PMC9219400; doi:10.1186/s12889-022-13663-2)
Supplement: Supplementary file 2 — Additional file 2. Detailed Data Extraction. [file 12889_2022_13663_MOESM2_ESM.docx]

**Additional File 2: Detailed data extraction**

| **Author, Year** | **Country, Disaster, Year, Length of Disaster** | **Study Design** | **Funding Source (Type)** | **Organization(s)** | **Time between disaster onset and study** | **Target Audience**  **Program Type**  **Description of Strategies/Program/Services**  **Duration of Strategy/Program/Services Post-emergency** | **Main Findings** |
| --- | --- | --- | --- | --- | --- | --- | --- |
| **Abara et al., 2014** | United States, Chlorine spill, 2005, 1 day | Descriptive | NR | South Carolina Department of Health and Environmental Control  University of South Carolina | NR | **Target Audience**: General population  **Program Type(s)**: Chronic disease management; Environmental health and safety  **Description**:   - State public health agencies and local leaders established community coalition directed at recovery process - Coalition activities included townhall meetings and training workshops to address community members’ questions and issues. Subsequent recovery steps were made collaboratively between state public health agencies and community coalition. - Establishment of community health tracking registry. - Health screenings conducted by community volunteers and public health workers - Public health practice transitioned to public health research three years after disaster.   Community Based Participatory Action Research was used to engage local citizens in recovery efforts in identifying and addressing community-identified health and environmental concerns  **Duration**: Ongoing | - Increased knowledge of community members related to environmental health risks of chemical spill - Improvements to poor water and sewer systems - Grants supported the retraining of former millworkers and others in Hazardous Waste Operations and Emergency Response - Establishment of a pulmonary diagnostic laboratory and health resource center |
| **Acosta et al., 2018**  **2018** | United States, Hurricane Sandy, 2012, 2 days | Cross-sectional | Assistant Secretary of Preparedness and Response at the US Department of Health and Human Services (Government) | New York Department of Health and Mental Hygiene  Community NGOs | 1 year | **Target Audience**: General population  **Program Type(s)**: Case Management; Medical Care; Community Liaison Services  **Description**:   - Community-based organizations (CBOs) in partnership with public health departments, provided a variety of services during disaster recovery, most commonly case management, medical care, home care, housing services and community liaison services.   **Duration**: 2 years | - CBOs provided public health post-disaster services including health services, home care, housing, mental health, and social services. - During recovery, CBOs expanded scope of services beyond core services offered during non-disaster times - CBOs were providing services 2 years after the storm and majority were planning to continue in the coming year. - CBOs operating in most-affected boroughs had a higher rate of tie formation (community partnership) than those working in less impacted areas. - Positive association found between organization’s number of connections and the number of types of impact that could be achieved. - Partnerships made during a disaster response are less durable, underscoring importance of establishing partnerships early through pre-established agreements. - Partnership facilitators: strong organizational leadership, history of collaboration among partners and shared interest in rebuilding the community. |
| **Centres for Disease Control and Prevention, 2013** | Haiti, Earthquake, 2010, The primary event lasted only a few seconds followed by 52 aftershocks over the course of two weeks | Descriptive | CDC and partners (Government, private, NGO) | The Haitian Ministry of Public Health and Promotion  CDC | Immediate | **Target Audience**: General population  **Program Type(s)**: Immunizations; Infectious diseases; Sexual health; Nutrition; Maternal/Infant health; Parenting and child development  **Description**: The HSRP provided technical expertise on: Epidemiology; Project management; Nutrition; Surveillance; Monitoring and evaluation; Multi-lingual capacity; Laboratory; Vaccine preventable diseases; Diplomacy; Water, sanitation, and hygiene; Tuberculosis; Logistics; Geographic information systems (GIS); Maternal and child health; Procurements; Informatics and reproductive health.   - Laboratory:   - Provided technical assistance, training, and funding to support the Haitian public health laboratory network to increase capacity and strengthen laboratory management.   - Developed a national specimen referral network - Surveillance:   - Established surveillance systems and the capacity to maintain and expand these systems. - Epidemiology:   - Established the Field Epidemiology Training Program (FETP)   - Provided training, collaborative project implementation, and ongoing technical assistance with Geographic Information Systems (GIS) - Vaccines:   - Established a temporary cold chain and supported the development of the infrastructure and expertise for a long-term, sustainable cold chain.   - Conducted a national MMR vaccination campaign and a survey to evaluate the campaign's effectiveness - Water, Sanitation, and Hygiene:   - Supported the department of Sanitation in the creation and training of Potable Water and Sanitation Technicians for the Communes, the creation of national guidelines and targeted communication campaigns.   - Assisted with the Cholera outbreak response - Lymphatic Filariasis (LF):   - Provided drug treatment for LF   - Trained MSPP and other partners to conduct the LF Transmission Assessment Survey - Tuberculosis:   - Assisted with establishing a self-contained laboratory and developed standard procedures for the diagnosis of TB - Maternal & Child Health:   - Designed and implemented a Violence Against Children Survey (VACS)   - Worked to increase access to emergency obstetric care, antenatal care and postnatal care, family planning, prevention of mother to child transmission of HIV, cervical cancer screening, and sexually transmitted infection diagnosis and treatment.   - Supporting the development of a Maternal Death Surveillance and Response System. - Malaria:   - Helped to procure rapid diagnostic tests (RDTs) and revise the national malaria diagnostic and case management policies - Rabies:   - Provided financial and technical assistance to improve laboratory diagnostic capacity and surveillance, risk assessment and decision-making and development of binational guidelines for rabies control and prevention - Nutrition:   - Conducted a national survey on malnutrition   - Supported the implementation of a National Sentinel Nutrition Surveillance System.   **Duration**: Ongoing | - NA |
| **Craddock et al., 2016** | United States, Hurricane Irene, 2011; Hurricane Sandy, 2012, Approximately 12 hours per storm | Qualitative | Uniformed Service University (Government funded academic institution) | Local Public Health Units  State/Provincial Agencies  FEMA  NGOs | 1 year | **Target Audience**: Organization staff (incident commander, emergency manager, public health director, mental health director, hospital emergency manager, the chiefs of fire and police, elected officials, and leaders of active volunteer organizations)  **Description**: NR  **Program Type(s)**: Emergency response and preparedness  **Duration**: NR | The respondents’ advice fell into 5 main categories:   - Planning and evaluation   - Plan for recovery   - Plan for infrastructural failures   - Conduct regular needs assessments   - Do a gap analysis and evaluate programs - Education and training   - Train executives and elected officials   - Practice and exercise   - Be inclusive: consider public agencies not “traditionally” involved in disaster preparedness and response.   - Understand health impacts   - Be ready to communicate with the public   - Be familiar with available federal assets   - Learn from others: Read other communities’ post-disaster plans for recovery   - Create a culture of preparedness - Fundraising and donations management   - Emphasize financial preparedness   - A donations management plan is essential from the beginning   - Document everything - Building relationships   - Establish and communicate realistic expectations of staff   - Develop relationships with public service organizations   - Build a volunteer management system.   - Recruit both local and nonlocal volunteers.   - Leverage the private sector   - Find a mentor   - Convene a long-term recovery committee: Immediately hold regularly scheduled recovery-planning meetings   - Maintain contact with vulnerable populations   - Plan for long-term case management - Disaster behavioural health   - Be sensitive to the psychological impacts of recovery   - Plan for mental health care needs - Promote self-care |
| **Fitter et al., 2017** | Haiti, Earthquake and Cholera Outbreak, 2010, Ongoing at the time of study publication | Descriptive | NR | Haitian Ministry of Public Health and Population  CDC  PAHO/WHO  Community NGOs | NR | **Target Audience**: General population  **Program Type(s)**: Immunizations; Infectious diseases; Maternal/Infant health  **Description**:   - Coordinating group for both local and international organizations, (initially led by PAHO/WHO then by MSPP), defined high-level public health recovery goals. - Recovery efforts focused on the surveillance, diagnosis, treatment, and prevention of cholera as well as efforts geared towards malaria, tuberculosis, HIV/AIDS, and maternal health   **Duration**: NR | Recovery efforts aligned with WHO’s EPHS Framework. This framework can provide guidance on where to focus efforts for success.    **Assessment**   - Monitor health (e.g., strengthening disease surveillance and laboratory capacity). - Diagnose and investigate (e.g., improve laboratory capacity for TB, malaria diagnosis, response plans for cholera flareups)   **Policy development**   - Inform, educate, empower (e.g., health education/promotion) - Mobilize community partnerships (e.g., local water committees) - Develop policies (e.g., develop national policy/procedures on TB testing)   **Assurance**   - Link to /provide care (e.g., vaccination campaign, practitioner training on cholera treatment) - Assure competent workforce (e.g., strengthening public health workforce capacity and establishing new programs. - Evaluate (e.g., evaluate immunization campaigns)   **Cross-cutting across all core functions**   - Research (e.g., vaccine effectiveness and coverage studies included in oral cholera vaccination efforts). - Systems management (e.g., strengthen management capacity) |
| **Geiger, D., Harborth, L. & Mugyisha, A., 2020** | Uganda, Ebola, 2018, 2 years | Descriptive | None | Uganda Red Cross Society | NR | **Target Audience**: Government staff & decision-makers, health care workers and NGO Staff  **Program Type(s)**: Infectious diseases  **Description**: Seven key lessons for managing long-term public health emergencies such as pandemics:   - centralised pooling and management of resources - engagement of local communities - continuously recruit and train staff - adjusted working patterns to prevent staff exhaustion - cooperation of involved agencies with security for enforcing measures - revision of funding frameworks - use of GPS data to identify population movement patterns.   **Duration**: NA | - NA |
| **Kennedy et al., 2019** | United States, Hurricane Harvey, 2017, 1 week | Qualitative | University of Colorado Natural Hazards Center (Government-funded academic institution) | Local Public Health Unit | 3 months | **Target Audience**: Local health department staff  **Program Type(s)**: Emergency response and preparedness  **Description**:  Visioning Recovery for Resilience   - Majority of respondents did not report participation in the development or implementation of a community-wide vision for a more resilient, and sustainable community post-disaster - Reactive approaches to recovery: provision of resources and addressing needs as identified   Planning   - Pre-planning more commonly conducted: activities focused on response and short-term recovery minimal planning on intermediate/long-term recovery   Post-event planning   - Formalized structure or group responsible for post-event recovery planning less common   Activity Implementation   - Minimal evidence of local health departments leveraging the recovery process to meet pre-disaster goals. - Most described intermediate/long-term essential recovery activity reported was health and safety inspections (e.g., at restaurants and schools).   Assessments   - Most reported mechanism for assessing community status post-disaster was through collection of anecdotal and informal information about community needs; surveillance monitoring of health outcomes   **Duration**: NR | - NA |
| **Kodish et al., 2019** | Guinea and Sierra Leone Ebola, 2014, 2 years | Qualitative | UNICEF (NGO) | Non-Governmental Organization  United Nations | 3 years | **Target Audience**: General population  **Program Type(s)**: Nutrition  **Description**: Organizational factors and lessons learned to facilitate nutrition response during outbreak:   - political will and policy - increased funding for nutrition - food assistance programs - nutrition sensitization and messaging to communities - establishment of treatment centres - enhanced coordination/establishment of food security network - increased staffing, capacity building - improved logistics - better nutrition monitoring and counselling - hygiene assistance - psychosocial counselling - community level actions and use of standard operating procedures - surveillance system for monitoring malnutrition   **Duration**: NR | - NA |
| **Lee et al., 2009** | Burma Civil Conflict, 2008, NR | Descriptive | NR | Karen Department of Health and Welfare  Backpack Health Worker Team  Global Health Access Programme | NR | **Target Audience**: Remote Villagers  **Program Type(s)**: Infectious diseases  **Description**: Teams conducted regular village visits and included:   - Trained lay community people providing malaria diagnosis, treatment, education, and referral services via regular house visits   **Duration**: NR | - Increased healthcare workforce and capacity |
| **Miller et al., 2018** | Guinea, Sierra Leone and Liberia , Ebola, 2014, 2 years | Mixed Methods | Government of Sweden (Government) and the Rockefeller Foundation (Private) | Local Public Health Unit  NGOs | ~2 years | **Target Audience**: General population  **Program Type(s)**: Immunizations; Infectious Diseases; Nutrition; Maternal/Infant Health; Parenting & Child Development  **Description**:   - Services provided by community health workers varied by country, district, and implementing partner. - Most common services delivered included:   - integrated community case management   - community sensitization and health promotion   - screening for childhood undernutrition   - childhood immunization   - distribution and promotion of insecticide-treated bed nets   - referrals for at-risk children and pregnant or postpartum women. - Most CHWs not implementing full package of services.   **Duration**: NR | - Decline in maternal, newborn, and child health service provision due to weak service delivery, confusion over policy, and the overwhelming nature of outbreak. However, many community health workers remained active in communities and were willing to continue providing services. - Clear directives facilitated community health worker provision - Service effectiveness was limited by lack of supply chain and supervision |
| **Melgaard et al., 2005** | Thailand, Earthquake and Tsunami, 2004, 1 day | Descriptive | NR | WHO | 5 months | **Target Audience**: International, national and local public health professionals  **Program Type(s)**: Emergency response and preparedness  **Description**: Strategies to better support existing healthcare system and contribute to health reform during recovery and reconstruction:   - needs assessment - coordination of recovery strategy: include long-term vision, government policies - filling gaps: appropriate resource allocation; performance indicator development and evaluation to assess recovery strategy - capacity building: involvement of local stakeholders with overall consistent goals/priorities at national level (systematic approach)   **Duratio**n: NR | - NA |
| **Quinn et al., 2008** | United States, Hurricane Katrina, Hurricane Rita and Hurricane Wilma, 2005, 1 month | Descriptive | Funds from the National Center on Birth Defects and Developmental Disabilities, CDC (Government) | Organization of Teratology Information Specialists’ (OTIS)  CDC | 1 week | **Target Audience**: Pregnant and Lactating Women  **Program Type(s)**: Maternal/Infant Health  **Description**:   - OTIS is a non-profit network of toll-free, telephone-based counselling services in hospitals, universities, and health departments - Activities included:   - national phone routing system and hurricane support page added to the web site   - Educational fact sheets and question-and-answer format summaries (English and Spanish) - Informational flyers with availability of services and information on hurricane-related exposure concerns (English and Spanish). Printed copies were distributed through public health, community, and service agencies.   **Duration**: 6 months | - Engagement: 33 hurricane-related calls and web site accessed 3,783 times. - Identified needs of pregnant and breastfeeding women during disasters and emergencies for future planning:   - Develop awareness of need for availability of information services for pregnant and lactating women in the event of a public health crisis.   - Incorporate mechanisms for systematic and rapid identification of concerns into disaster preparedness plans   - Incorporate mechanism for streamlining access to informational services   - Develop capacity to immediately activate an emergency response plan; include plans to provide services for several months after event. - Plan for collection and evaluation of real-time data concerning exposures to pregnant and lactating women and exposed infants |
| **Rogers et al., 2015** | Liberia, Ebola, 2014, 2 years | Single-Group Pre-test/Post-Test | Partners in Health Liberia (NGO) | Partners in Health | 1 year | **Target Audience**: TB, HIV and Leprosy patients  **Program Type(s)**: Infectious diseases; Nutrition  **Description**: Community health worker program:   - **Clinic immersion:** integration of CHW through mentoring, relationship building, patient navigation links - **Field immersion:** home visitation, referrals, patient and contact tracing - **Community engagement and health promotion:** dramas, radio, door-to-door - **Integrated approach to patient care:** addressing social determinants of health: Provision of transportation, food, social assistance   **Duration**: 6 months | - TB treatment coverage increased from 7.7% to 43.2% (p < 0.001) post-intervention and lost to follow-up rates decreased from 9.5% to 2.1% (p = 0.003). - ART treatment coverage increased (p = 0.03), with patient retention improving from 63.9% to 86.1% (p < 0.001); a 6.0 percentage point decrease in HIV lost to follow up was also observed (p = 0.21). |
| **Russell et al., 2018** | United States, Hurricane Sandy, 2012, 2 days | Mixed Methods | New York State Superstorm Sandy Social Services Block Grant (Government) | Rockaway Wellness Partnership  Visiting Nurse Service of New York | 2 years | **Target Audience**: Community-dwelling Adults  **Program Type(s)**: Chronic Disease Management; Nutrition; Alcohol, Drug & Tobacco Use; Physical Activity  **Description**:   - Health coaches assessed participants, set goals, and provided counselling on different health topics (e.g., nutrition, stress reduction), - Community health workers connected clients to health care and community services, including free or reduced cost primary care, educational and job-readiness programs, food pantries, entitlements, and legal assistance.   **Duration**: 2 years | - Goal Setting: Between 4%-35% of participants set goals according to various health topics - Referral rates**:** Ranged between 12%-29% for different medical, dental, and community programs - Increased self-rated health (M=2.8 baseline vs M=3.4 post-intervention; p<.001) and use of health care services - 73% reported increased confidence in health goal achievement - 99% would recommend the program to family and friends |
| **Schnall et al., 2019** | United States (US Virgin Islands), Hurricane Irma and Hurricane Maria, 2017, 2 weeks | Descriptive | NR | US Virgin Islands Department of Health | 7 weeks | **Target Audience**: General population and regional health department staff  **Program Type(s)**: Emergency response and preparedness  **Description**:   - Community Assessments for Public Health Emergency Response (CASPER) is an epidemiological technique designed to provide household-based information about a community’s needs in a timely, inexpensive, and representative manner. - Two CASPERs were conducted: November 2017 to gauge the hurricane response, and February 2018 to measure recovery.   **Duration**: 4 months | - Recovery CASPER helped to identify the health status (e.g., chronic health and mental health conditions) and multifaceted needs of residents and captured the progression of the recovery process. - Sharing of CASPER results with response partners facilitated coordinated, interagency recovery efforts. |
| **Stoto et al., 2013** | United States, H1N1, 2009, 1.5 years | Qualitative | CDC (Government) | Local Public Health Unit  State/Provincial Agencies  CDC | NR | **Target Audience**: Local, state and federal health department staff  **Program Type(s)**: Infectious diseases; Emergency response and preparedness  **Description**: Strategies:   - resource mobilization and legal authority: emergency declaration to elicit increase in funding, authority, and service contracts, and staff coordination - complexity of vaccine distribution and administration: group prioritization; community partnership development - balancing emergency response and routine operations: tiered volunteer system; hire per diem staff to develop vaccination response teams in rural areas - communication and coordination among the many independent actors in the public health system   **Duration**: NR | NA |
| **Tappis et al., 2020** | Yemen, Civil Conflict, 2015, Ongoing | Case report | Centre for Global Child Health at the Hospital for Sick Children (Private) | Local Public Health Unit  State/Provincial Agencies  NGOs | 3 years | **Target Audience**: Women and children residing in 3 Yemeni cities; Sana’a City, Aden and Taiz  **Program Type(s)**: Infectious diseases; Sexual health; Nutrition; Maternal/Infant health; Parenting and child development  **Description**:   - Humanitarian response coordination to provide basic health services at facilities, deploying mobile clinics, outreach teams, community health worker, and volunteer networks, short-term project cycles - Prioritization of health services for women and children: focus on outbreak response and treatment of acute malnutrition - Availability, retention, qualified health workers: contracting staff, task shifting, in-service and on-the-job training   **Duration**: 1 year | - Service delivery challenges and coping mechanisms included: insecurity, the politicization of aid, the availability, retention and motivation of qualified health workers, damaged and neglected infrastructure, unavailability of supplies. - Barriers to seeking care included: Access and affordability and distrust and lack of demand |

NGO: Non-governmental organization; TB: Tuberculosis; HIV: Human Immunodeficiency Virus; CHW: Community health worker; ART: Antiretroviral therapy; NR: Not reported; NA: Not applicable; CDC: The Centres for Disease Control and Prevention; PAHO: Pan-American Health Organization; WHO: World Health Organization; MSPP: Ministry of Public Health and Population (Haiti); EPHS: Essential public health services; AIDS: Acquired immunodeficiency syndrome; CBO: Community-based organization; OTIS: The Organization of Teratology Information Specialists’; COVID-19: Coronavirus disease 2019; GPS: global positioning system; UNICEF: United Nations International Children’s Emergency Fund; CASPER: Community Assessment for Public Health Emergency Response; HSRP: Health systems reconstruction program (CDC); FEMA: Federal Emergency Management Agency; LF: Lymphatic Filariasis; MMR: Measles, mumps, rubella
